# Integrated circuits based on broadband pixel-array metasurfaces for generating data-carrying optical and THz orbital angular momentum beams

**DOI:** 10.1515/nanoph-2023-0008

**Published:** 2023-03-15

**Authors:** Alan E. Willner, Xinzhou Su, Hao Song, Huibin Zhou, Kaiheng Zou

**Affiliations:** Department of Physics and Astronomy, University of Southern California, Los Angeles, CA 90089 USA; Department of Electrical and Computer Engineering, University of Southern California, Los Angeles, CA 90089, USA

**Keywords:** metasurface, multiplexing, orbital angular momentum, photonic-integrated circuits

## Abstract

There is growing interest in using multiple multiplexed orthogonal orbital angular momentum (OAM) beams to increase the data capacity of communication systems in different frequency ranges. To help enable future deployment of OAM-based communications, an ecosystem of compact and cost-effective OAM generators and detectors is likely to play an important role. Desired features of such integrated circuits include generating and detecting multiple coaxial OAM beams, tunability of OAM orders, and operation over a wide bandwidth. In this article, we discuss the use of pixel-array–based metasurfaces as OAM transmitters and receivers for mode division multiplexing (MDM) communications in near-infrared (NIR) and terahertz (THz) regimes.

## 1 Introduction

There has been much interest in space division multiplexing (SDM) by transmitting multiple data channels over the same spatial medium. Specifically, SDM has the potential to significantly increase the aggregate data capacity and spectral efficiency (i.e., bits/s/Hz) of a communication system by simultaneously transmitting multiple independent data-carrying beams [1–4]. A subset of SDM is mode division multiplexing (MDM), in which each channel is carried by a unique mode from a basis set of orthogonal spatial modes [2–5]. The orthogonality between different modes implies that multiple independent data-carrying beams with different mode orders can be multiplexed at a transmitter aperture, spatially copropagate, and be demultiplexed at a receiver aperture with little inherent channel crosstalk.

**Figure 1:**
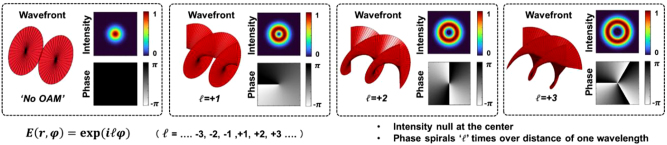
The beam profile of OAM modes (wavefront, intensity and phase, and OAM order *ℓ* = 0, 1, 2, and 3). OAM beams with nonzero mode order have helical wavefronts and ring-like intensity profiles [8].

Orbital angular momentum (OAM) is one example of the modal set that consists of multiple orthogonal modes and can be used for MDM systems. As shown in Figure 1, an OAM beam can be typically characterized by (i) a phase front that “twists” as it propagates, (ii) a mode order *ℓ* which is the amount of OAM and represents the number of 2π phase changes in the azimuthal direction, and (iii) an intensity profile that has a ring shape with a central null (i.e., vortex) [6–9]. OAM can be considered a subset of Laguerre–Gaussian (LG) modes [6–9].

Various demonstrations of communication systems using OAM-based multiplexing have been shown for electromagnetic (EM) and mechanical waves across different frequency regimes (e.g., optical, radio wave, millimeter wave, terahertz (THz), and acoustic wave) [10–19]. Many of these demonstrations have used large components at both the transmitter and receiver for generating and detecting the specific OAM value of a data-carrying beam. Likely challenges for future potential deployment of OAM-based systems include the development of compact and cost-effective OAM devices. Generally, the desired functions of integrated OAM emitters/detectors might include the capabilities to (i) tune the mode order of the generated OAM beam, (ii) emit an OAM beam over a broad spectral bandwidth, and (iii) generate multiple coaxial OAM beams [20–38].

Importantly, metasurface-based integrated devices, which are composed of multiple subwavelength-patterned structures to shape the amplitude and phase of light, have shown interesting features, including a compact footprint, broad bandwidth, and high efficiency [39–42]. Specifically, various metasurface-based subwavelength structures have been utilized to (a) shape a single input Gaussian beam to an OAM beam with high OAM order, high mode purity [43], and/or broad bandwidth [44] and (b) convert different input Gaussian beams with different input angles or polarizations to multiple coaxial OAM beams with different OAM orders [44]. Interestingly, there might be the potential for broadband pixel-array metasurface structures to generate multiple OAM beams that specifically have tunability when considering multiple inputs with tunable phase delays.

In this paper, we review recent experimental demonstrations of using pixel-array–based metasurface mode converters for OAM-based MDM communication systems. Such a design can be applied to OAM beams in different frequencies (e.g., optical and THz). For a free-space optics (FSO) system, the metasurface structures can be used as OAM transmitters and receivers. The generated/detected OAM mode order can be tunable using additional phase controllers [45–47]. For a free-space terahertz (THz) system, the lower carrier frequency leads to a stronger demand for fractional bandwidth (i.e., the absolute bandwidth divided by the center frequency). The broad bandwidth of the pixel-array–based OAM emitter can help to achieve high data rate wireless THz communications [48].

## 2 Background of OAM-based communications

**Figure 2:**
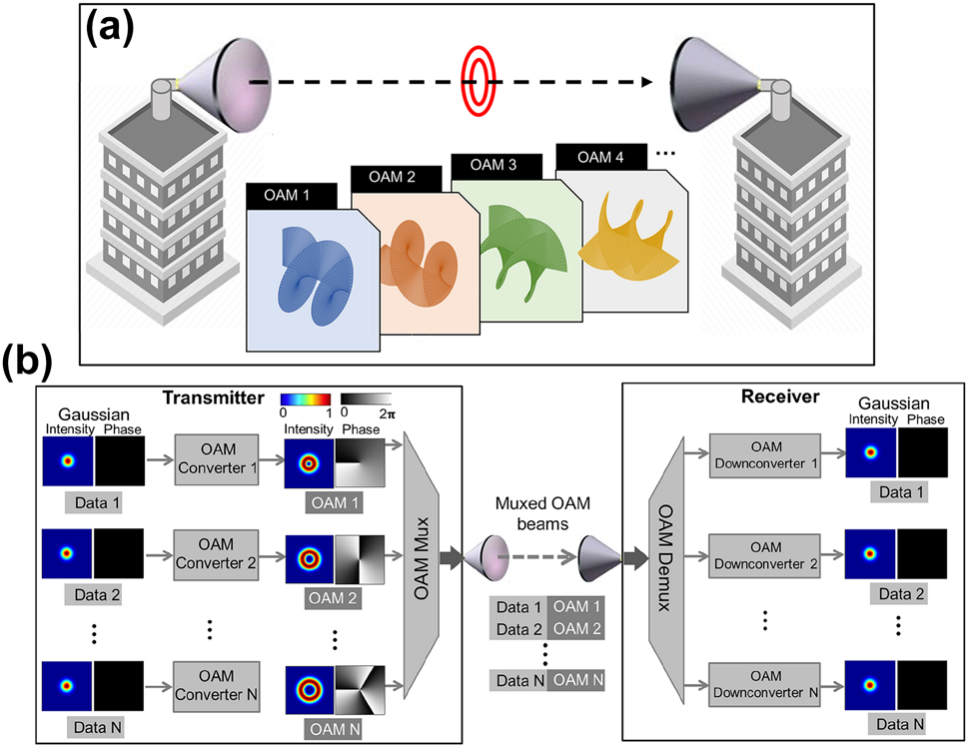
Scheme diagram of MDM communication systems using OAM multiplexing. (a) Multiple OAM beams can be coaxially transmitted within a single aperture pair [16]. (b) Each orthogonal OAM beam can carry an independent data stream, thereby multiplying the data capacity by the number of multiplexed beams [10].

The orthogonality between OAM beams with different orders enables OAM-based MDM communication systems. Figure 2 shows an example of an OAM-based MDM system. Each OAM beam with a different OAM order can carry a different independent data stream. Different data-carrying OAM beams are spatially multiplexed and propagated coaxially through the same data channel [13]. At the receiver, due to the mutual orthogonality between modes, multiplexed data channels can be demultiplexed efficiently with little inherent crosstalk [13]. As a result, the system’s data capacity and spectral efficiency (i.e., bit/s/Hz) can be enhanced by a factor of *N* when *N* different OAM beams are multiplexed. Moreover, OAM multiplexing is generally compatible with other multiplexing techniques, such as wavelength/frequency division multiplexing (WDM/FDM) and polarization division multiplexing (PDM) [13]. Nonetheless, OAM beams can be considered as a subset of the two-dimensional (2D) LG_*ℓ*,*p*_ modal basis. An LG beam is characterized by two spatial modal indices: (i) azimuthal index *ℓ*, which is the OAM order, and (ii) radial index *p*, where *p* + 1 is the number of concentric intensity rings [7, 8, 49]. Theoretically, 2D LG modes with different *ℓ* and/or *p* values are also orthogonal to each other and suitable for MDM systems. Compared with an MDM system using a one-dimensional (1D) OAM beam, utilizing a 2D modal set could potentially provide a larger set of orthogonal data channel choices for multiplexing [50].

We note that OAM can be used in the encoding of individual symbols in a single-beam communication system. Orthogonal OAM modes can create a large alphabet for data symbols, and a single beam can be encoded on different OAM modes within discrete time windows [51, 52]. Although the devices described in our paper can be used for encoded OAM systems, we discuss in this paper mainly OAM-based MDM communication systems.

**Figure 3:**
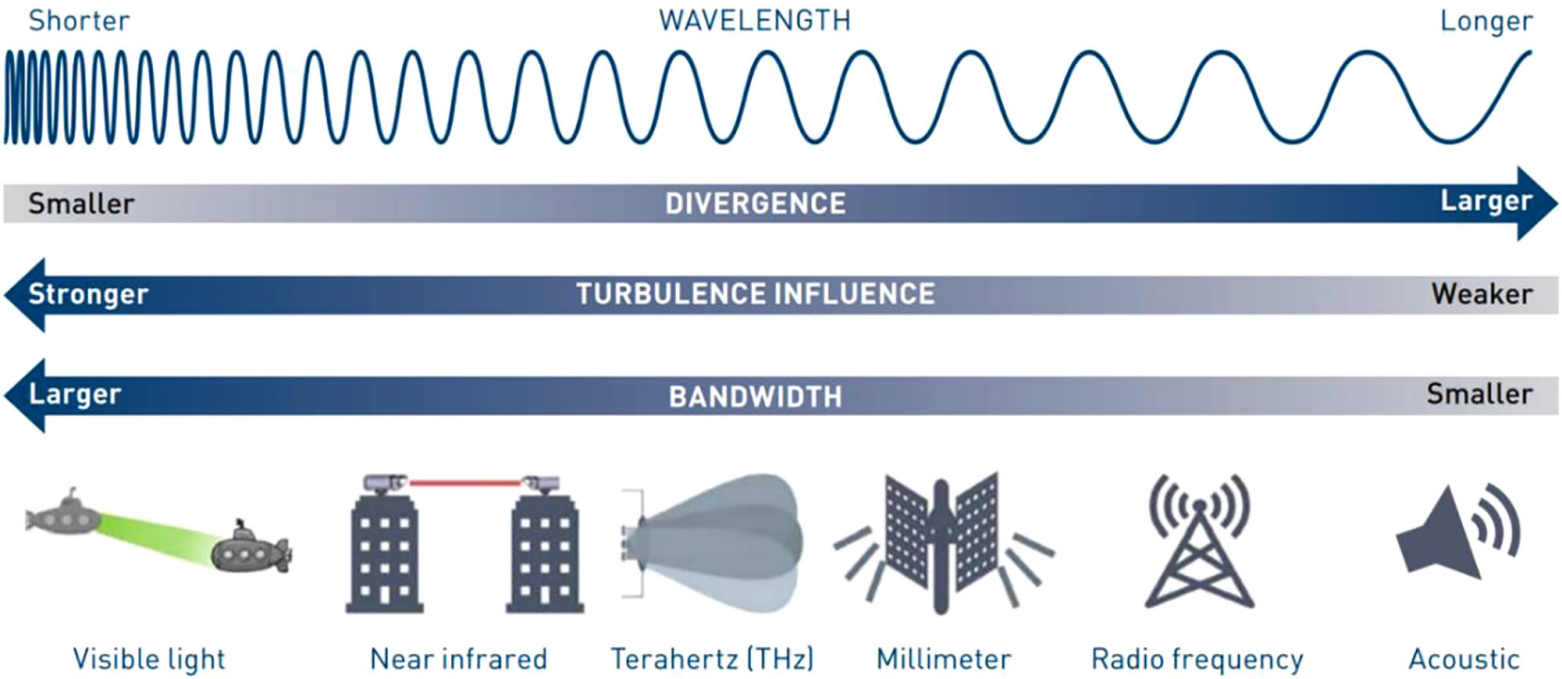
Communications systems for different types of electromagnetic and mechanical waves with different frequencies [66].

As a general property of many different electromagnetic and mechanical waves, OAM can help enhance aggregate data capacity and spectral efficiency for systems in different frequency regimes (e.g., radio wave, millimeter wave, THz, NIR, mid-infrared (MIR), visible light, and acoustic wave). As shown in Figure 3, there tends to be a trade-off between the beam divergence and interaction with matters for free-space beams with different carrier frequencies: (i) higher frequencies have lower beam divergence and (ii) lower frequencies tend to have lower interaction with matter. In this article, we review integrated devices for OAM-based systems mainly in optical and THz ranges as two examples. Some general features of the two frequency bands are summarized as follows:

(i) Optical wave has higher carrier frequency compared to lower frequency bands for wireless communications. Generally, the higher frequency provides larger bandwidth and lower beam divergence. The lower divergence leads to a lower probability of intercept while making the pointing and tracking harder. In addition, atmospheric turbulence tends to have stronger distortion on optical beams due to the higher frequency of optical waves. For FSO at 1550 nm, fiberoptics components are mature and available, and systems can take advantage for high-speed communications.
(ii) THz wave has smaller spectral bandwidth compared to the optical wave. However, it still has larger bandwidth and lower beam divergence when compared to millimeter waves [53] and lower beam degradation due to atmospheric turbulence when compared to optical waves [54, 55]. In particular, the spectral band in the 300-GHz carrier wave frequency region is low in atmospheric absorption and turbulence effects [54]. Taking advantage of these issues, several demonstrations have been reported of free-space THz communication links [56–63]. Interestingly, THz communications have garnered interest for potential deployment in ultra-high data rate communications for wireless local area networks (WLANs) (where the IEEE 802.15.3d standard has been established) and 6 G systems [64, 65].

Due to the general properties of EM waves, similar designs could be used for different frequency ranges. However, different frequency ranges might require different base technologies, such as different materials, bandwidths, and sizes.

### 2.1 Photonic-integrated circuits for OAM generation and detection

For ease of description, we will first discuss the issue of integrated devices for OAM generation and detection in the optical frequency regime.

**Figure 4:**
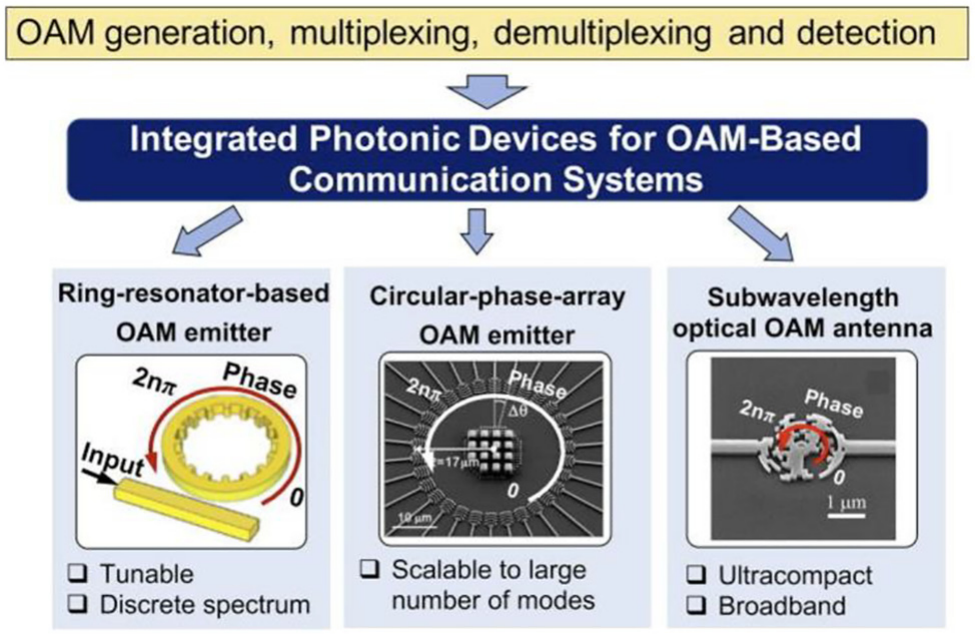
Different integrated photonic devices for OAM generation/detection and (de)multiplexing [23, 27, 29].

Recently, there has been significant research on designing and fabricating photonic-integrated circuit (PIC) devices to generate OAM beams, thereby advancing the deployment of OAM systems with efficient, cost-effective, and compact technologies [20–38]. There are certain desirable features for integrated OAM devices, including low insertion loss, fast tunability, a large number of modes, efficient mode conversion, and a wide wavelength range [44, 67]. Various novel designs of integrated photonic devices have been utilized for OAM-based communication links (Figure 4), including (a) ring resonator–based OAM emitters/receivers embedding angular grating structures with a periodic modulation of the refractive index in the azimuthal direction, which support OAM beams with tunable OAM orders [21, 25, 29]; (b) circular phase-array OAM emitters/receivers with multiple circular optical antennas to generated/receiver multiple OAM beams [22, 27, 28]; and (c) subwavelength optical OAM antenna with a relatively compact and specifically designed metasurface to achieve broadband OAM generation/detection [23, 32, 33, 37, 38].

For a circular phase-array OAM emitter, multiple antennas could generate a large number of modes. However, the relatively large footprint might limit its application in large-scale integration. In addition, the compact micro-ring resonator–based structure could also be limited by its discrete wavelength spectrum. To achieve broadband OAM generation with a relatively compact structure, metasurface-based structures have been utilized, such as pixel-array–based metasurface OAM antenna and free-space metasurface phase plate. These metasurfaces show various interesting properties, including design flexibility, OAM mode tunability, and broadband OAM generation/detection in OAM-based communication systems [23, 43, 68], [69], [70].

### 2.2 Concept of broadband pixel-array–based metasurfaces for optical OAM beams

Pixel-array–based OAM emitter, as a type of metasurface-based structure, has the advantage of broad working bandwidth. Additionally, this type of structure is generally easy to be integrated with other PIC components. In this article, we will discuss mainly about pixel-array–based OAM devices.

**Figure 5:**
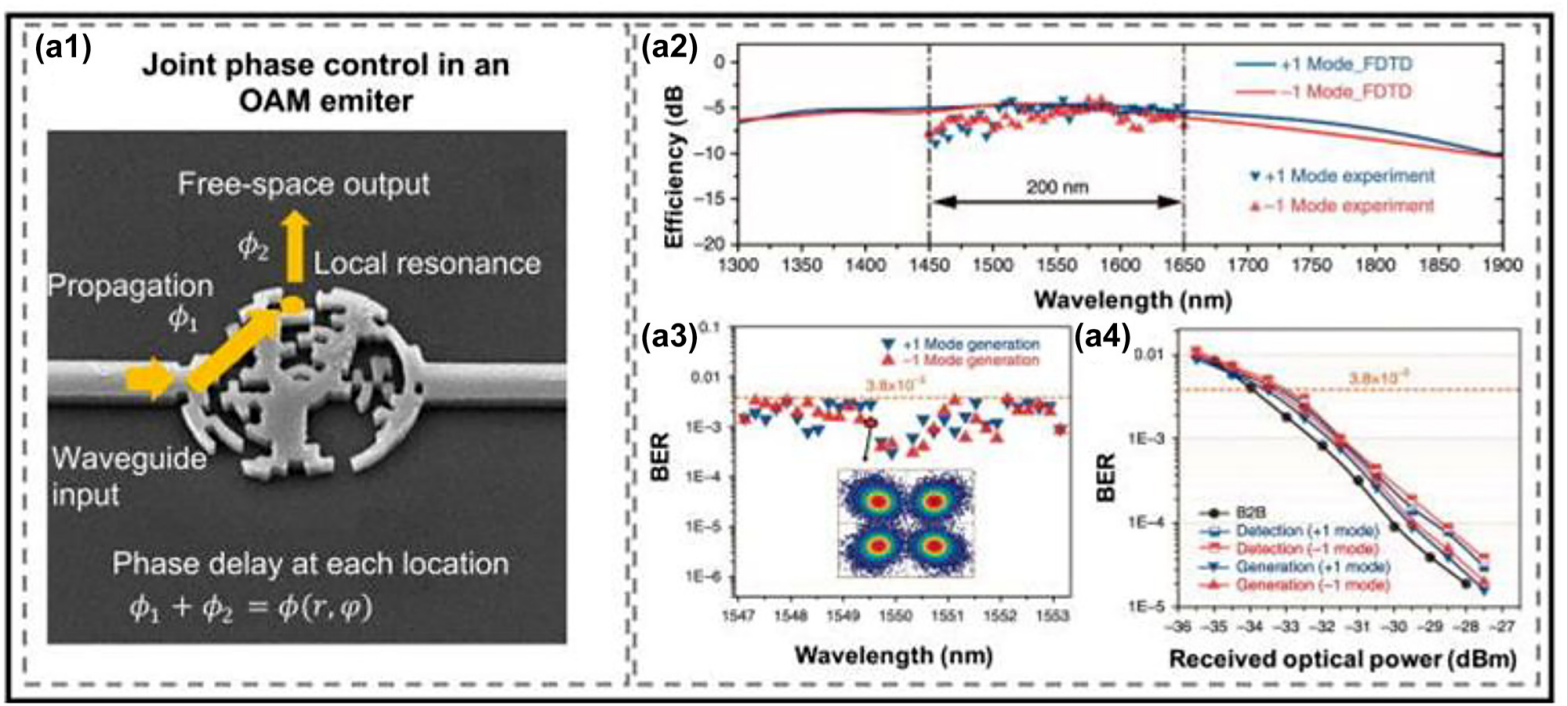
OAM generation utilizing the pixel-array–based metasurfaces OAM emitter. (a1) The phase delay at each location consists of both the propagation-induced phase *ϕ*_1_ and the pixel-induced phase *ϕ*_2_. (a2) Conversion efficiency of the subwavelength OAM emitter at different wavelengths. (a3) The measured bit error rates (BERs) of the OAM +1 and −1 modes in an MDM and WDM link (30 wavelengths and two OAM modes). (a4) BER performance of the cases using this device for detection and generation at 1550 nm. B2B, back to back [23].

An example of a pixel-array–based mode converter with two input ports used as an OAM emitter is shown in Figure 5. The generated beam profile is jointly controlled by the propagating path length and different pixels at different locations. In general, the resulting phase delay at each location $\phi \left(x,y\right)$ consists of the propagation-induced phase (*ϕ*_1_) and the pixel-induced phase (*ϕ*_2_). To design the pixel-array–based OAM mode converter, the generated phase profiles from the left port (*ϕ*_left_) and the right port (*ϕ*_right_) are required to be as follows [23]:

(1) $$\left\{\begin{array}{c} \phi_{\text{left}}={\phi_{1}}_{\text{left}}+{\phi_{2}}_{\text{left}}=\ell_{\text{left}}\theta \;\\ \phi_{\text{right}}={\phi_{1}}_{\text{right}}+{\phi_{2}}_{\text{right}}=\ell_{\text{right}}\theta \\ , \end{array}\right.$$

where *θ* is the azimuthal angle, and *ℓ*_left_ and *ℓ*_right_ are the mode orders of the generated OAM beams from the left and the right ports, respectively. Subsequently, the beam generated by feeding from different ports (left/right) has different azimuthal 2π phase changes and thus has a different OAM order. It should be noted that the analytical solution to these equations is difficult to obtain [23, 71]. Alternatively, an optimization algorithm can be utilized to design the distribution of the pixels to jointly control the spatial phase profiles [23, 71].

One example of optimization algorithms can be direct binary search [71]. In each iteration, input ports are excited and the output fields from the mode converter are captured to evaluate the converted modal purity. Given the normalized output field *E*_output_, the corresponding modal purity *C* can be calculated as

(2) $$\begin{array}{c} C={\left|\iint E_{\text{output}}E_{\text{Target}}^{*}\right|}^{2}, \end{array}$$

where *E*_Target_ is the normalized ideal field of the target mode. Subsequently, the mode converter can be iteratively optimized by (i) randomly switching the state of the pixels one at a time and (ii) maximizing the total modal purity of different input ports.

Based on the designed broadband pixel-array–based OAM emitter, a free-space link [23] has been demonstrated, combining MDM and WDM. Two inputs, each has 30 frequency channels, are fed into the chip to generate two OAM channels (*ℓ* = +1 −1). Each channel carries a 20-Gbit/s quadrature phase-shift keying (QPSK) signal, and a total 1.2 Tbit/s capacity is achieved with the two multiplexed OAM beams, as shown in Figure 5(a3) and (a4).

In general, the bandwidth and conversion efficiency of the metasurface-based integrated OAM devices are mainly dependent on each subwavelength local resonator [23, 32, 38, 45]. Since the subwavelength resonators tend to have a relatively small Q factor and do not have a specific resonant wavelength, the structure can achieve high mode purities over a broad bandwidth [23, 32, 38, 45]. In addition, the conversion efficiency of the structure can be enhanced by optimizing the etch depth of subwavelength elements and adding reflective layers [32].

## 3 Pixel-array–based tunable metasurfaces in NIR optical systems

The pixel-array–based metasurface provides a broad working bandwidth [23, 32]. Furthermore, one key desirable feature of pixel-array–based OAM generation/detection would be the tunability of the OAM order of each channel. Interestingly, that can be achieved with external phase control.

In this section, we will focus on recent experimental demonstrations of optical communication systems using the broadband pixel-array–based metasurface device, which could generate multiple OAM beams with tunable mode orders using tunable phase controllers [46, 47].

### 3.1 Concept

**Figure 6:**
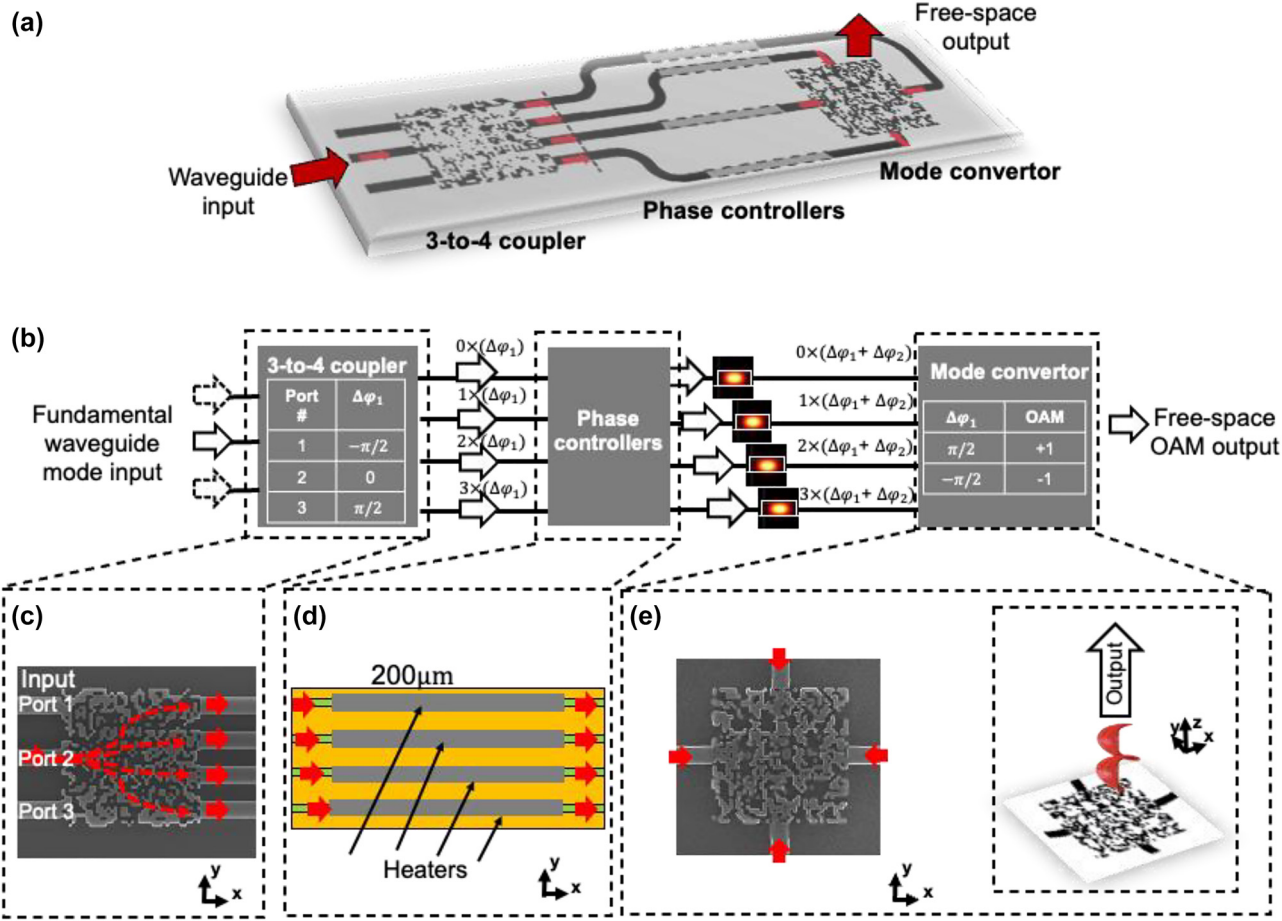
Structure of the pixel-array–based OAM metasurface emitter. (a) Main concept. (b) The emitter is composed of a 3-to-4 coupler, four tunable phase controllers, and a mode converter. The OAM order of the output beam is dependent on the phase delay of the waveguides. This phase delay is related to both the phase delay induced by the 3-to-4 coupler and the tunable phase controllers, respectively. (c) Scanning electron microscopy (SEM) image of the pixel-array–based 3-to-4 coupler. (d) Integrated thermal phase controllers. (e) SEM image of the pixel-array–based mode converter [46, 47].

The concept of tunable OAM generation by a pixel-array–based metasurface OAM emitter [46] is shown in Figure 6. The OAM emitter is comprised of a 3-to-4 coupler, four phase controllers, and a mode converter. Specifically, the tunable OAM emitter (Figure 6(b)) works as follows:

(i) The fundamental waveguide mode is fed into one input port and gets coupled into four output waveguides with an equal power distribution by the 3-to-4 coupler; meanwhile, the 3-to-4 coupler is designed to introduce a specific phase delay (*k* − 1)Δ*φ*_1_ to the fundamental waveguide mode in the *k*th waveguide (*k* = 1, 2, 3, 4).
(ii) From the 3-to-4 coupler to the mode converter, an additional phase delay (*k* − 1)Δ*φ*_2_ is added to the *k*th waveguide (*k* = 1, 2, 3, 4) by the integrated phase controller; furthermore, the length of the connecting waveguides between the coupler and the mode converter is equal in the design to mitigate the bandwidth reduction due to the waveguide length mismatch [72].
(iii) Subsequently, the designed OAM mode converter coherently combines and converts the multiple waveguide inputs with the accumulated phase delay (Δ*φ* = Δ*φ*_1_ + Δ*φ*_2_) into a single free-space beam-carrying OAM.

The embedded tables in Figure 6(b) show the transformation functions of (i) the port number and the phase delay Δ*φ*_1_ for the designed 3-to-4 coupler and (ii) the accumulated phase delay (Δ*φ*_1_ + Δ*φ*_2_) of the input coherent waveguide modes and the OAM order of the output free-space beam for the designed mode converter, respectively. By tuning the phase delay Δ*φ*_2_, the OAM order of the generated beam could be tuned. For example, by feeding port 2 (Δ*φ*_1_ = 0) and tuning the phase delay from Δ*φ*_2_ = −*π*/2 to Δ*φ*_2_ = *π*/2, the OAM order of a single beam could be tuned between *ℓ* = −1 and *ℓ* = +1, respectively. Moreover, by feeding port 1 (Δ*φ*_1_ = −*π*/2) and port 3 (Δ*φ*_1_ = *π*/2) of the coupler simultaneously, two multiplexed OAM beams (*ℓ* = −1 and +1) could be generated. Importantly, as there is no specific resonant wavelength, the designed structure could achieve the OAM generation of the same mode order over a relatively broad bandwidth [23].

The pixel-array structure of the 3-to-4 coupler and mode converter are designed using finite-difference time-domain (FDTD) simulation. In addition, the direct binary search (DBS) algorithm is iteratively run to optimize the pixel-array structure based on the specific objective functions (e.g., the mode purity of generated OAM beam) [32, 45]. Specifically, the DBS algorithm of the mode converter works as follows: (i) *initialization*: an initial pattern is set as a 2-D grating structure with a period of 600 nm, and the FDTD simulation is run to get the initial value of the objective function; (ii) *binary search*: the material of a randomly chosen pixel is changed from silicon to silica, or inversely. Such toggle introduces a perturbation into the last structure and a new value of the objective function is calculated by running FDTD simulation. The new structure would be kept if the value increased; otherwise, the last structure would be restored; (iii) *iteration*: such binary search, including the random toggle, FDTD simulation, objective function calculation, value comparison, and structure decision are operated iteratively until the maximum step number is reached. A similar design procedure is also applied for the design of the 3-to-4 coupler. It should be noted that there are some other algorithms (e.g., the fabrication-constrained topology optimization and the genetic algorithm), which might be more efficient for designing the in-plane couplers and out-of-plane mode converter with the desired transformation function [23, 33].

Such a pixel-array–based OAM emitter is fabricated in an electron-beam lithography process. Both pixel-array–based structures are composed of 40 × 40 silicon/silica pixels with a pixel size of 100 × 100 nm and a total pixel-based metasurface area of 4 × 4 μm, as shown in Figure 6(c) and (e). The thicknesses of the top oxide cladding layer, the silicon layer, and the bottom buried oxide layer are 2.2 μm, 220 nm, and 2 μm, respectively. Four 200-μm titanium–tungsten heaters (Figure 6(d)) are fabricated above the waveguides as phase controllers.

### 3.2 Experimental demonstration of a single OAM beam with tunable OAM orders

**Figure 7:**
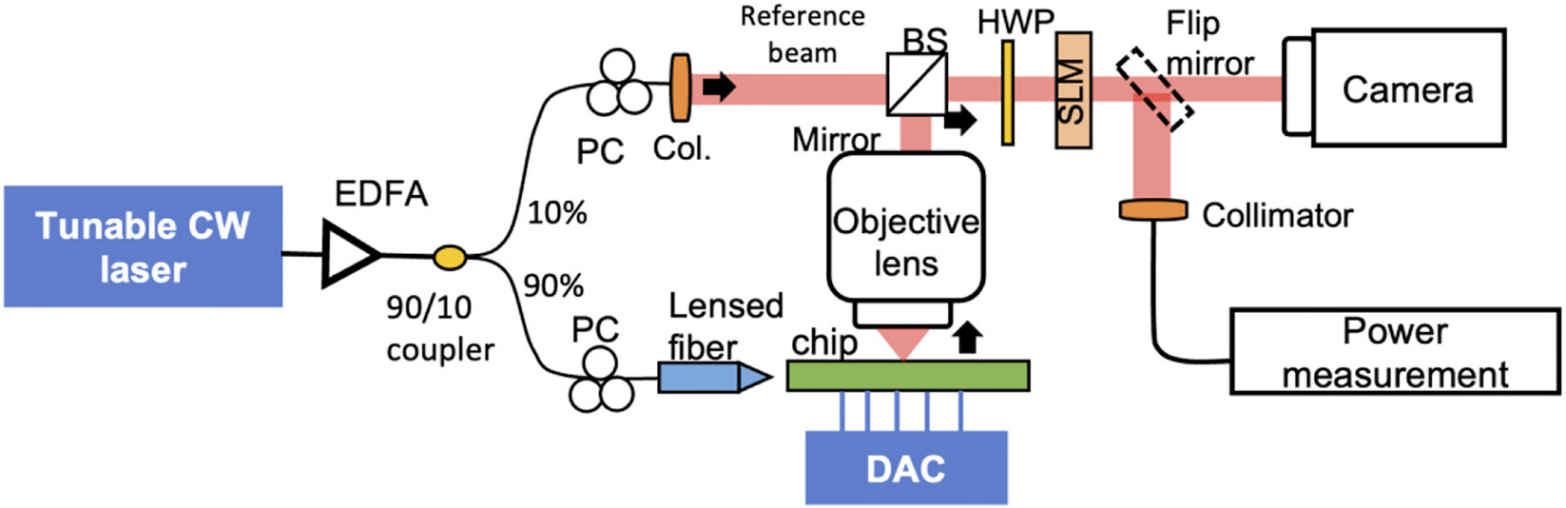
Experimental setup of free-space beam characterization for the tunable OAM emitter. EDFA, erbium-doped fiber amplifier; Col, collimator; BS, beam splitter; HWP, half-wave plate; DAC, digital-to-analog converter; SLM, spatial light modulator [46].

The experimental setup for characterizing the generated beam by the tunable OAM emitter is shown in Figure 7. A CW laser is amplified and then split into two arms. One is sent to the metasurface OAM chip, and the other is sent to generate a reference Gaussian beam. A lensed fiber is used to couple the light to the chip. As the edge coupling of the chip is polarization sensitive, polarization controllers are used to align the polarization of the input beams. The off-chip bias control (i.e., digital-to-analog converter) is applied to tune the integrated heaters of the chip. It should be noted that such bias control could both compensate for the phase error induced by the waveguides [31] and also tune the generated OAM order. To find the optimum bias, each of the four bias voltages (V1, V2, V3, V4) is coarsely swept in steps of 1 V first and then finely tuned in steps of 0.1 V by monitoring the intensity profile of the output beam. At the output, the generated beam is coupled by an objective lens (NA = 0.4) and propagates through ∼0.5 m of free space. At the receiver, a free-space half-wave plate is used to align the polarization of the emitted OAM beams with that of the spatial light modulator (SLM). To measure the OAM modal power distribution of the generated beam, the optical power is collected and measured in the fiber by loading the spiral phase patterns on the SLM.

**Figure 8:**
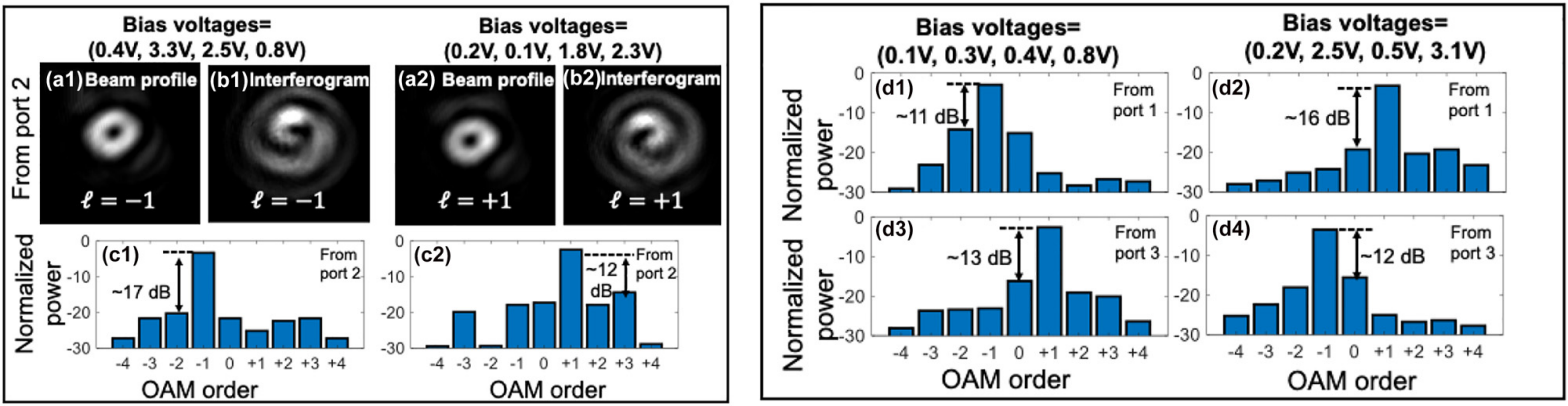
Measured (a1 and a2) beam profiles, (b1 and b2) interferogram, and (c1 and c2) modal power distribution of a single beam when port 2 is fed. Measured modal power distribution of a single beam when (d1 and d2) port 1 and (d3 and d4) port 3 are fed. The wavelength of the CW laser is 1550 nm. The OAM order is tuned by applying different bias voltages [46].

The measured mode purity of the generated beam under different bias voltages is shown in Figure 8. Applying different bias voltages and selecting port 2 as an input port, the beam profiles, and interferogram are shown in Figure 8(a1, a2) and (b1, b2), respectively. The interference pattern of the generated beam with a coherent Gaussian reference beam is characterized. The “twisting” phasefront of the output beam indicates the OAM order of the generated OAM beam. The opposite “twisting” directions indicate that the OAM order is changed by tuning the bias voltages. Furthermore, the modal power distribution of the output beam is measured as shown in Figure 8(c1, c2). Under the two different tuning conditions, the highest power coupled from the desired mode to the neighboring modes is <∼−17 dB and <∼−12 dB for OAM beams of *ℓ* = −1 and *ℓ* = +1, respectively. The conversion efficiency of the mode converter is ∼−10 dB. In addition, the generated beams when feeding port 1/3 under different bias voltages are characterized as shown in Figure 8(d1–d4). By tuning the applied voltages, the corresponding OAM order when feeding port 1/3 could also be tuned. Under the same bias voltage, different OAM orders can be generated by feeding different ports (i.e., ports 1 and 3). For all the cases mentioned, the intermodal power coupling is <−11 dB. This intermodal power coupling could be induced by the nonideal power split of the coupler, imperfect phase control, and residual undesired scattered light from the OAM emitter.

**Figure 9:**
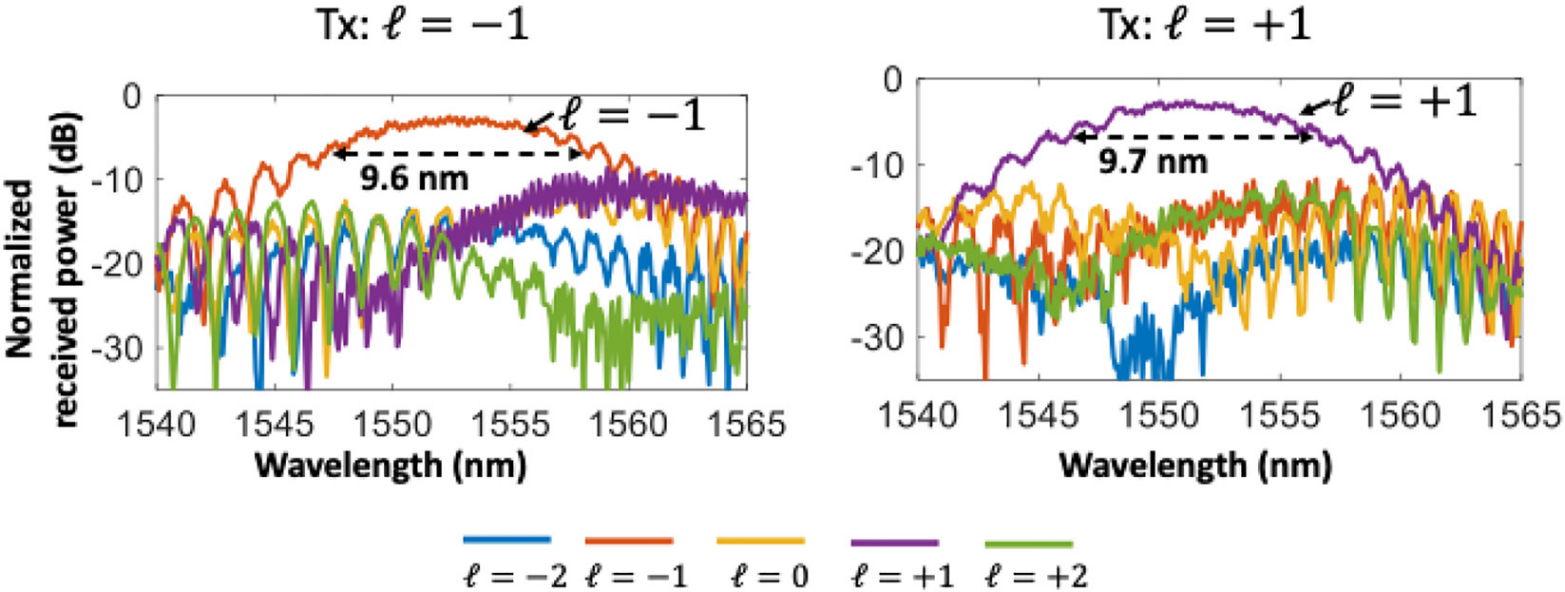
Measured modal power distribution at different wavelengths of a single beam with a tunable OAM order of (a) OAM −1 or (b) OAM +1. As proof of the concept, port 2 is selected as the input port [73].

The measured bandwidth of the pixel-array–based metasurface OAM emitter is shown in Figure 9. By sweeping the input wavelength from 1540 to 1565 nm, the bandwidth performance of the OAM generation is measured. The measured 3 dB bandwidth of the desired mode is ∼9 nm, as shown in Figure 9. The bandwidth could potentially be limited by (a) wavelength-dependent amplitude and phase error induced by the fabrication error and (b) the length mismatch of the connecting waveguides between the coupler and the emitter.

### 3.3 Mode and wavelength multiplexed links using the tunable metasurface OAM chip

**Figure 10:**
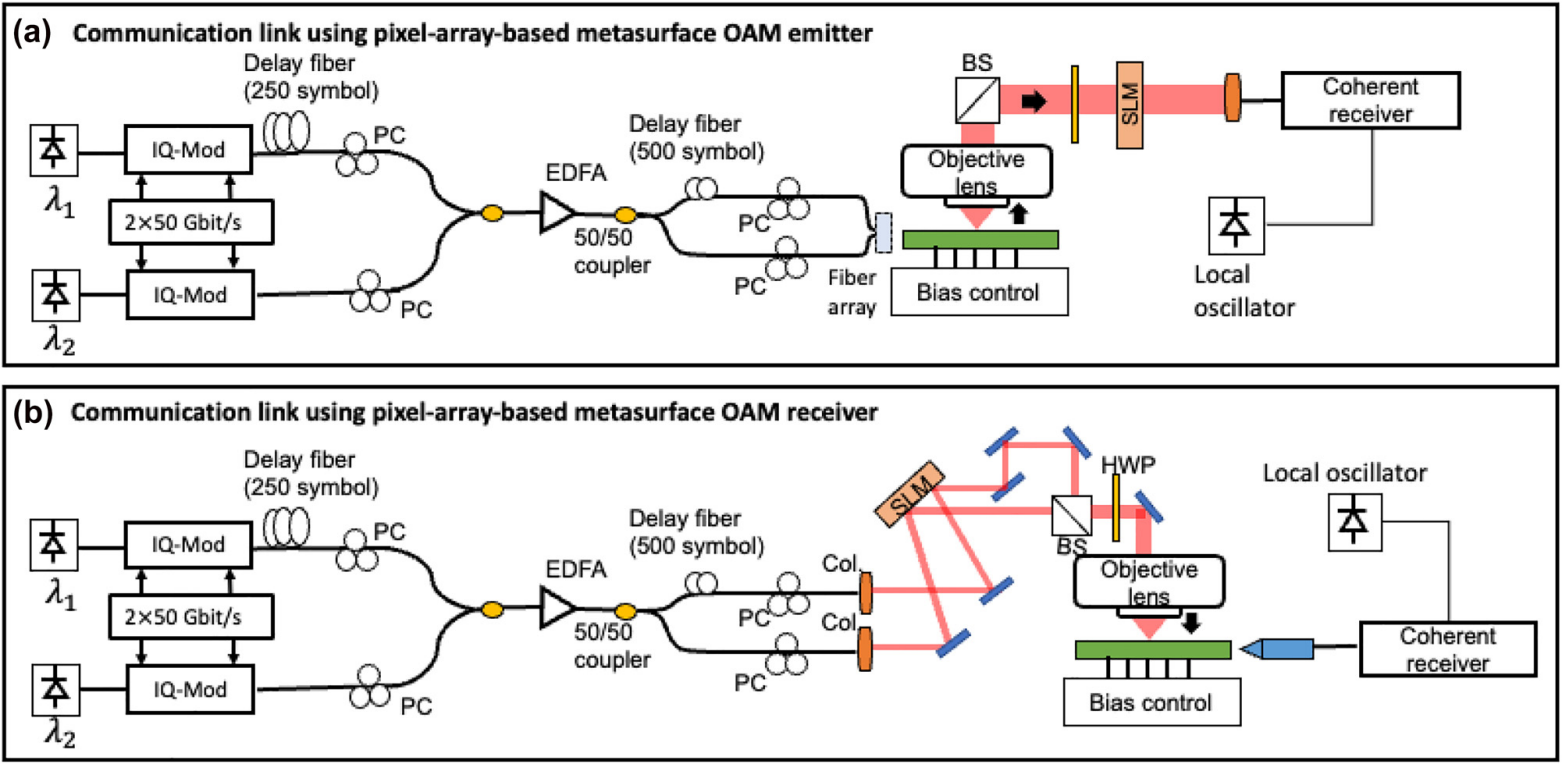
Experimental setup of the OAM-multiplexed and WDM links using (a) the pixel-array–based metasurface OAM emitter or (b) the pixel-array–based metasurface OAM receiver. Carrier wavelength *λ*_1_: 1550.9 nm, *λ*_2_: 1551.7 nm [46, 47].

The experimental setup of the communication link using the pixel-array–based metasurface OAM emitter is shown in Figure 10(a). Each WDM channel is first generated from a separate IQ modulator and combined. The polarization controllers are used for keeping the same polarization of the two 50-Gbaud WDM QPSK channels. Subsequently, the two WDM channels are amplified and split into two copies. They are decorrelated by passing through fibers of different lengths. Then, the two copies are respectively fed to port 1 and port 3 of the OAM emitter for generating multiplexed OAM beams (*ℓ* = −1 and *ℓ* = +1). By tuning the applied bias voltages, the OAM orders of the generated beams could be changed, and thus the corresponding data channels carried by the OAM beams can be changed. At the receiver side, the generated data-carrying OAM beams are demultiplexed by an SLM, and subsequently, the collected optical signal carried by the corresponding generated OAM beam is recovered by the coherent receiver.

Reversely, the pixel-array–based metasurface can also be used as an OAM receiver in a communication system, as shown in Figure 10(b). Two OAM beams with mode order of *ℓ* = −1 and *ℓ* = +1 are generated by sending the Gaussian beam through different phase patterns loaded on different areas of the SLM. Subsequently, the two beams are (i) multiplexed using a beam splitter (BS), (ii) coaxially propagated in free space for ∼0.5 m, and then (iii) collected by the objective lens and coupled vertically into the chip. A half-wave plate is used before the objective lens to align the input polarization to the target polarization. By tuning the applied bias voltages, the data channel carried by one of the multiplexed OAM beams is sorted to the designated port, received by the lensed fiber, and sent to a coherent receiver.

**Figure 11:**
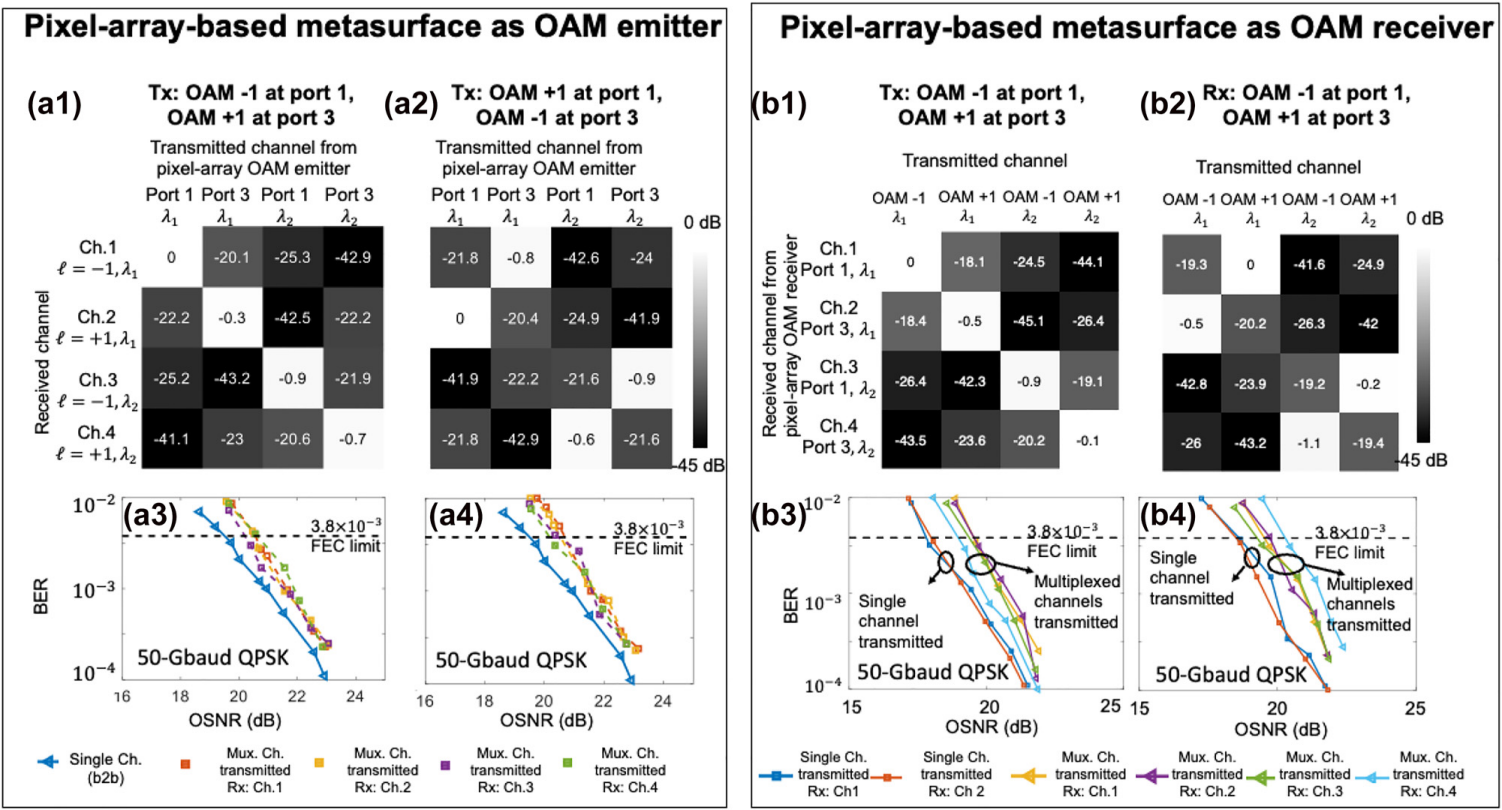
Experimental setup of the 400-Gbit/s OAM-multiplexed and WDM links using: (a) the pixel-array–based metasurface OAM emitter or (b) the pixel-array–based metasurface OAM receiver. Carrier wavelength *λ*_1_: 1550.9 nm, *λ*_2_: 1551.7 nm [46, 47].

A 400-Gbit/s OAM-multiplexed and WDM link is demonstrated utilizing the pixel-array–based metasurface as OAM emitter or OAM receiver, as shown in Figure 11. For the OAM emitter, the OAM order of the generated beams can be tuned by changing the bias voltages, and thus the corresponding data channels carried by the OAM beams can be switched. Figure 11(a1–a4) shows the measured interchannel crosstalk and BER performance of different WDM channels and different data channels carried by the OAM beams. Compared with the single channel carried by a single Gaussian beam (without going through the chip), there is a ∼1 dB OSNR penalty to achieve the BER of 3.8e-3 for the two WDM channels carried by either OAM *ℓ* = −1 or *ℓ* = +1. For the OAM receiver, the multiplexed channels are generated by the benchtop setup (i.e., SLM and BS) and free space coupled into the chip. The intermodal crosstalk is <−17 dB for both OAM-carried channels *ℓ* = −1 and +1 at the same carrier wavelength, as shown in Figure 11(b1 and b2). The measured crosstalk between different WDM channels is <−20 dB. Compared to the single-wavelength and single-OAM channel, there is a <2 dB OSNR penalty for all the OAM-multiplexed and WDM channels, as shown in Figure 11(b3 and b4). The OSNR penalty could be mainly due to the intermodal crosstalk induced by (i) the nonideal amplitude distribution and phase shift of the four waveguides inputs and (ii) the imperfect misalignment between the transmitter and the receiver [45, 74].

## 4 Pixel-array–based metasurfaces for THz systems

Similar to using PIC for photonic OAM-based systems, integrated circuits might also bring important value to OAM-based THz communications [75–78]. Reports of THz OAM-related integration include (a) transmissive or reflective metasurfaces that can convert free-space Gaussian beams to free-space OAM beams [79–82] and (b) integrated electronic circuits that can directly generate a free-space THz wave carrying OAM with tunable mode order, such that each bit can be encoded on different OAM modes at ∼ Mbit/s [83].

The similar design of pixel-array–based metasurfaces for OAM generation, detection, and (de)multiplexing in the NIR range could also be applied to THz frequencies. In this section, we review one example of using a silicon slab with pixel-array–based metasurface that generates multiple >Gbit/s OAM beams and multiplexes them together for transmission in the THz range [48].

### 4.1 Concept and design principles

The THz integrated OAM emitter can share a similar design principle as the pixel-array–based metasurface OAM converter. However, considering different material platforms and system configurations at different frequencies, special designs would be required for the THz systems:

(a) To achieve low-loss signal transmission, the fabrication of the devices uses a single-layer 200-μm-thick high-resistivity float-zone intrinsic silicon wafers with resistivity of >20 kΩ cm [77, 84]. The relative permittivity *ϵ*_si_ is estimated to be 11.68 and corresponding to a refractive index of $n_{si}=\sqrt{\epsilon_{si}}=3.417$ . Since the chip is only single-layer silicon, the design should also consider the mechanical handling of the device.
(b) For THz systems, the signal can usually be guided in hollow metallic waveguides. Thus, the THz integrated OAM emitter needs to be designed with coupling ports that can be inserted into the hollow waveguides, and couple data-carrying THz signals to the silicon slab.

**Figure 12:**
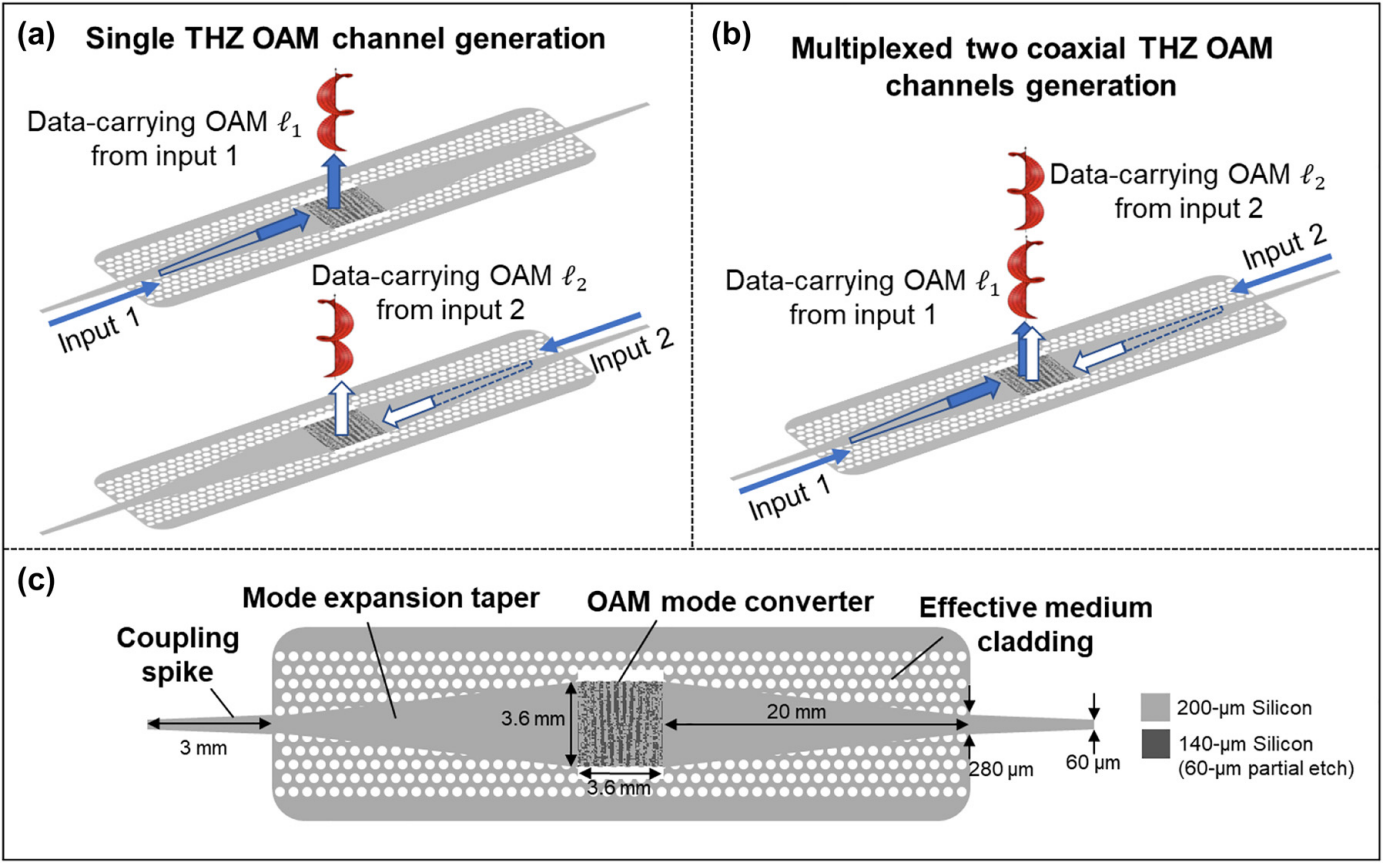
Concept of pixel-array–based THz integrated OAM emitter. (a) The integrated OAM emitter can generate a single data-carrying THz OAM beam with signal coupled from the input waveguide. (b) The emitter can be designed to generate two coaxially multiplexed OAM beams with different orders with signals coupled from different inputs. (c) Schematic diagram of the integrated OAM emitter [48].

Figure 12 shows the concept of a pixel-array–based THz integrated OAM emitter. The center of the device is a partially etched pixel-array–based metasurface OAM mode converter. As shown in Figure 12(a), with the signal coupled from the input waveguide, the mode converter can vertically emit a free-space OAM beam. Furthermore, the integrated OAM emitter can be designed so that with a signal from different input ports, spatially multiplexed OAM beams with different OAM orders can be emitted into the free space. Figure 12(b) illustrates an example of two coaxially multiplexed data-carrying OAM channels from two different inputs.

Figure 12(c) illustrates the three main regions of the device: (i) two input coupling spikes; (ii) two mode expansion structures made of adiabatic tapers surrounded by low-refractive-index effective medium cladding [84]; and (iii) a pixel-array–based OAM mode converter with an area of 3.6 × 3.6 mm^2^.

The following paragraphs introduce the major differences compared to the photonic-integrated OAM emitters, including (a) the effective medium and (b) the coupling spikes.

(a) **Effective medium:** As mentioned, mechanical support needs to be taken into consideration for such a single-layer device. Specially for this device, an effective medium made of a periodic dense hollow lattice is used to connect the mode expander and the mechanical supporting frame. The relative permittivity of the effective medium is estimated by the Maxwell-Garnett approximation [77, 84] to be:
(3) $$\begin{array}{c} \epsilon_{\text{eff}}=n_{\text{eff}}^{2}=\epsilon_{si}\frac{\left(\epsilon_{0}+\epsilon_{si}\right)+\left(\epsilon_{0}-\epsilon_{si}\right)\zeta_{d}}{\left(\epsilon_{0}+\epsilon_{si}\right)-\left(\epsilon_{0}-\epsilon_{si}\right)\zeta_{d}}, \end{array}$$
  where *ϵ*_0_ and *ϵ*_si_ are the relative permittivities of air and silicon, respectively, and *ζ*_*d*_ represents the fill factor of the air in the silicon. The value of the filling factor is dependent on the pattern of the array of holes. For a hexagonal lattice, the filling factors can be calculated as $\pi d^{2}/\left(2\sqrt{3}a^{2}\right)$ , where *d* is the hole diameter, and a is the lattice constant, that is, the distance between the centers of two adjacent holes. In this design, with *d* = 110 μm and *a* = 120 μm, the filling factor *ζ*_*d*_ is 0.76, and the index of the effective medium is thus around 1.6.
(b) **Coupling spikes:** For the 300 GHz THz wave, WR3.4 hollow metallic waveguides are often used to transport the THz signal. Typically, these kinds of hollow metallic waveguides support the fundamental transverse-electric (TE) mode. To couple the THz signal from the waveguides to the single-layer silicon chip, the coupling spikes have been demonstrated with a trapezoid shape. In this design, the spikes have widths of 60 μm and 280 μm, and a length of 3 mm. Nonetheless, it should be noted that for an integrated THz system, the coupling spikes are not necessary, and the mode converter could potentially be directly integrated with other components, such as the THz source and receiver [77].

**Figure 13:**
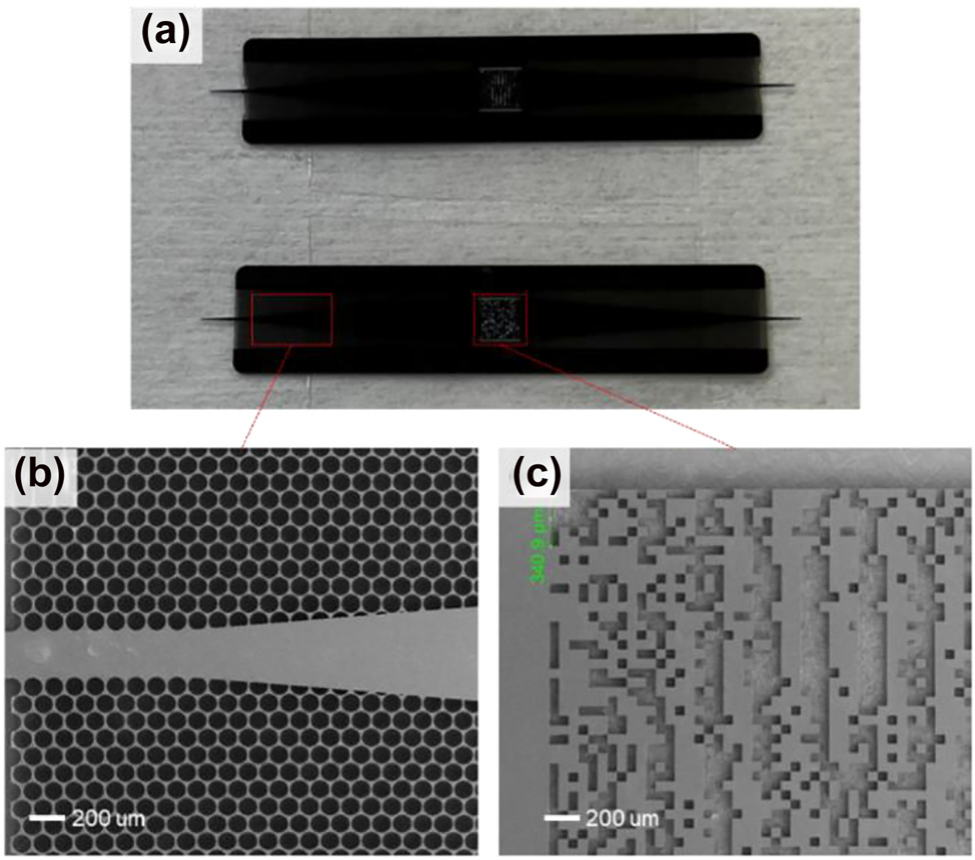
Pictures of the fabricated THz integrated OAM emitter. (a) Pictures of the whole structure. (b, c) SEM pictures of the silicon waveguides and pixel array structure [48].

Pictures of fabricated OAM emitters are shown in Figure 13(a), and the SEM pictures of the tapering structure, effective medium cladding, and OAM converter are presented in Figure 13(b, c).

### 4.2 Demonstration of a THz link using two multiplexed OAM beams

In this demonstration, at the transmitter (Tx), the THz data signal wave is generated using a photonic-assisted approach, while at the receiver (Rx), the THz signal is detected using an electronic approach. At the Tx, two continuous-wave (CW) lasers, with one of them modulated with data, are mixed in a positive-intrinsic-negative (PIN)-PD-based THz emitter to generate THz wave in the free space. The carrier frequency *f*_THz_ corresponds to the frequency difference Δ*f* between the two lasers. In order to couple the THz signal into the integrated emitter, a THz horn antenna with a hollow metallic waveguide is used to transfer the THz wave from the free space to the waveguide. At the Rx, to receive THz data-carrying OAM beams, spiral phase plates (SPPs) are first used to convert THz OAM beams to Gaussian beams. Subsequently, the received THz signal is down-converted to the intermediate frequency (IF) band by beating with a frequency multiplied radio frequency signal from an electrical local oscillator using a subharmonic down-converter. Subsequently, the IF data signal will be recorded with a real-time digital oscilloscope and processed for data information recovery and analysis.

**Figure 14:**
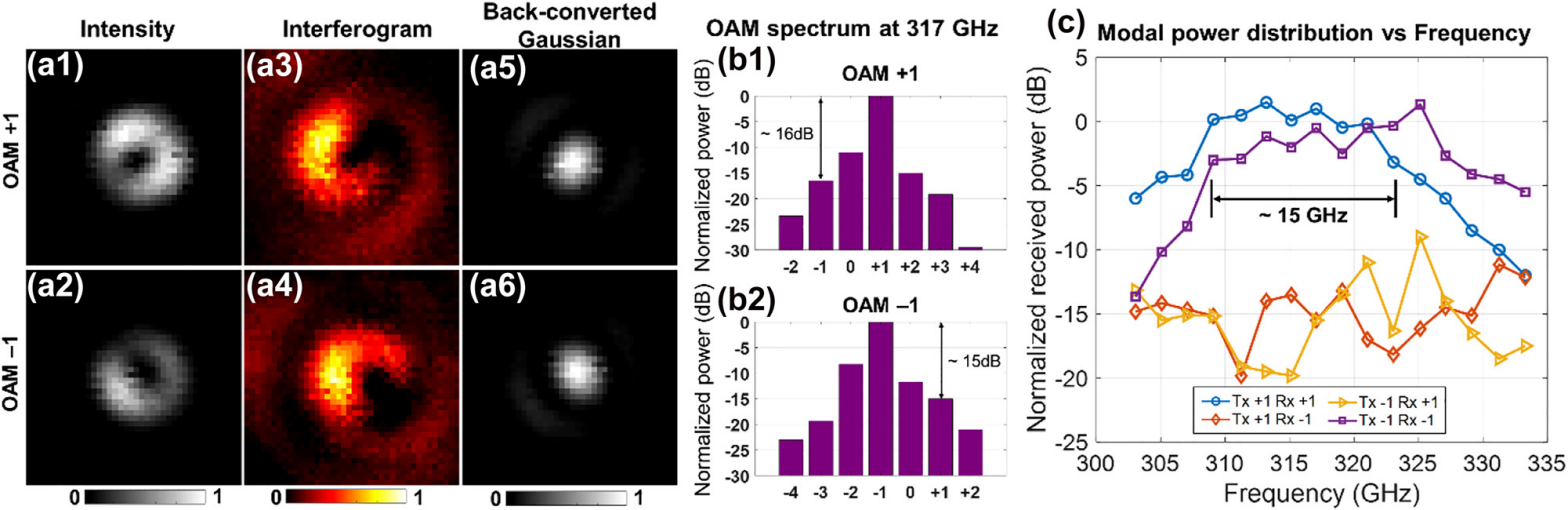
Experimental characterization results of the THz integrated OAM emitter. (a) Beam profiles for the generated OAM +1/−1 by the THz integrated OAM emitters ((a1, a2) intensity profiles, (a3, a4) interferogram with a THz Gaussian beam, and (a5, a6) back-converted Gaussian beam). (b) Modal spectra for OAM +1/−1 beams at the center frequency of the device ∼317 GHz. (c) Modal power distribution at different frequencies ranging from 303 GHz to 333 GHz [48].

To characterize the OAM beams generated by the integrated emitter, the intensity profile of the generated OAM beams is first measured using a CW THz input. In this case, the data modulation on the laser is switched off. As shown in Figure 14(a1, a2), the generated OAM ±1 beams have a ring-shaped profile and a diameter of ∼13 mm. In addition, the interferogram of the OAM beam with a Gaussian beam is presented in Figure 14(a3, a4). The input THz wave is power-split to make a coherent copy. The interferogram of OAM ±1 modes has one rotating arm and opposite rotating directions (clockwise or counterclockwise). The intensity profiles of back-converted Gaussian beams from the OAM beams are also investigated using corresponding SPPs (Figure 14(a5, a6)).

Figure 14(b1, b2) show the modal spectra of the OAM beams generated from the OAM emitter. The received power on different OAM beams is measured by changing the SPP mode order at the Rx. For the OAM +1/−1 modes, the power leaked to the neighboring modes is ∼−11 dB and ∼−9 dB, respectively. The crosstalk between the OAM +1 and −1 modes is ∼−16 dB at the center frequency of 317 GHz. Furthermore, the bandwidth of the device is characterized by a similar receiving setup. The received power of OAM +1/−1 modes is measured when transmitting OAM +1/−1 at different frequencies. Figure 14(c) shows that the integrated OAM emitter has a relative 3 dB power bandwidth of ∼15 GHz, and the channel crosstalk can remain <−10 dB within this band.

**Figure 15:**
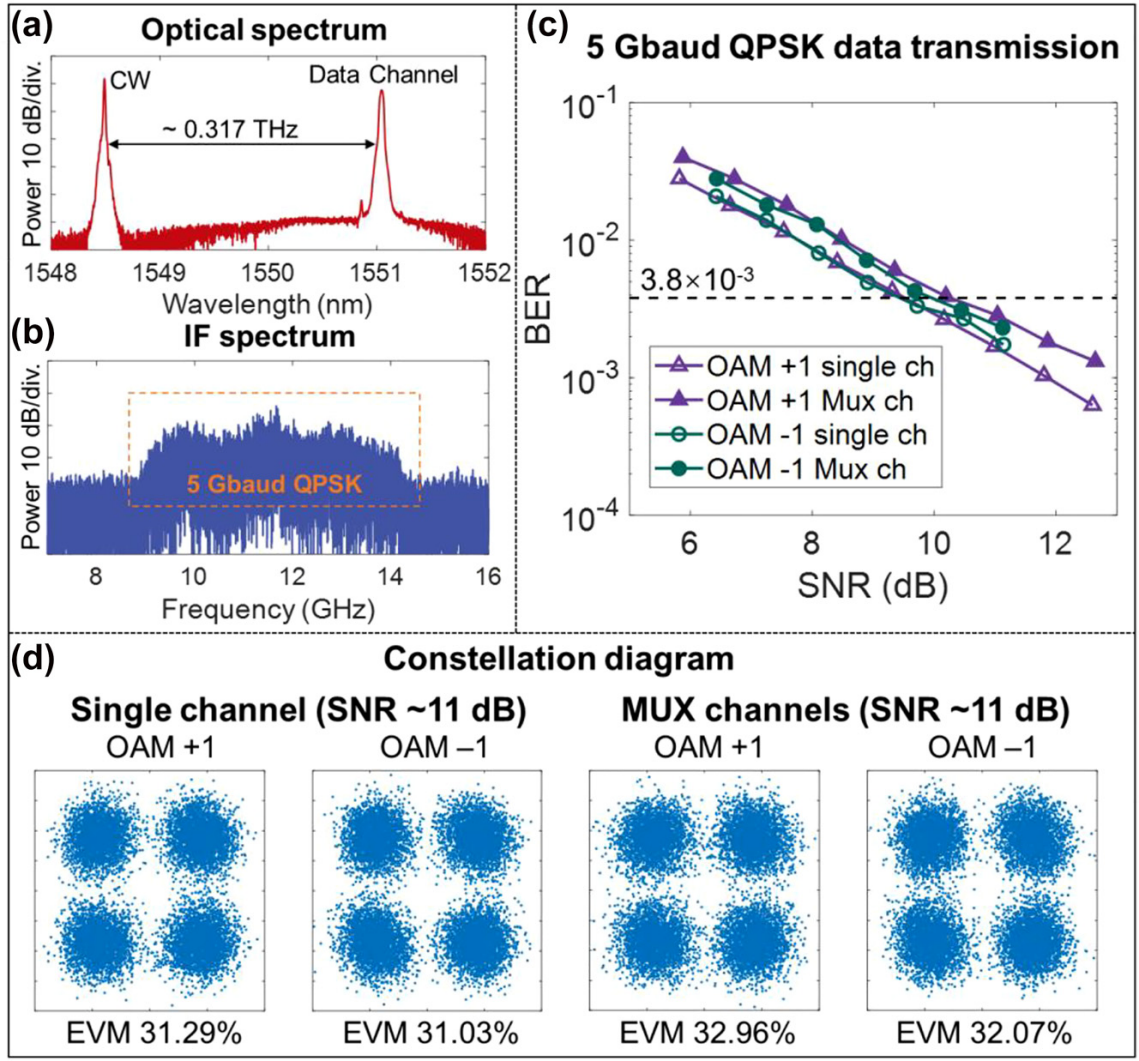
Experimental data transmission results. (a) Optical spectrum at the THz transmitter. (b) Electrical spectrum after down-conversion at the receiver. (c) Measured BER as a function of SNR for two multiplexed OAM channels with a 5-Gbaud QPSK signal for each channel. (d) The EVMs and constellation diagrams under an SNR of ∼11 dB for different cases [48].

The data transmission is subsequently evaluated using the integrated OAM emitter. Each OAM +1 and −1 beam carries a 5-Gbaud QPSK signal, and, in total, a 20-Gbit/s QPSK THz wireless link is achieved. Figure 15(a, b) show the optical spectra at the Tx and the electrical spectra after down-conversion at the Rx. The BER performance of the OAM +1/−1 channels at different SNRs is presented in Figure 15(c). Compared to single-OAM channels, there are ∼1 dB SNR penalties at the BER of 3.8 × 10^−3^ for the OAM-multiplexed THz link, due to the crosstalk from different OAM data-carrying channels. Figure 15(d) shows the corresponding constellations and measured error vector magnitudes (EVMs) for single or multiplexed data channels at an SNR of ∼11 dB. The constellation diagrams become more blurred, and the EVMs increase for the multiplexed OAM channels because of the interchannel crosstalk.

## 5 Summary and discussion

In summary, this paper discusses recent experimental demonstrations of integrated OAM (de)multiplexers using broadband pixel-array–based metasurfaces. Table 1 summarizes the experimental demonstrations discussed in this paper of different integrated pixel-array–based metasurfaces for various communication systems in different frequency ranges. Such kinds of OAM converters can be used for transmitters and receivers of OAM-based MDM communication systems. The generated/detected OAM mode can be switched using a tunable phase controller. Moreover, this kind of metasurface design can be used for integrated OAM converters for different frequency ranges, such as NIR and THz.

**Table 1:** Experimental demonstrations of different integrated pixel-array–based metasurfaces for various communication links mentioned in this paper.

Frequency	Metasurface	Number of	Channels, modulation	Bandwidth	Tunability	Ref.
region	function	mixed modes	format, & capacity			
NIR (1550 nm)	OAM mux/demux	2	400 Gbit/s QPSK (2 modes, 2 wavelengths)	∼9.5 nm	Yes	[46, 47]
THz (300 GHz)	OAM mux	2	20 Gbit/s QPSK (2 modes)	∼15 GHz	No	[48]

For future development of integrated OAM converters, there are still multiple challenges that might be interesting to address, including:

(a) **Mode orders:** A laudable goal might be to generate and/or detect OAM beams with higher orders and then (de)multiplex more modes. Potentially, a larger mode converter area with a smaller pixel size would likely support the larger modal fields of higher-order modes [85].
(b) **Number of modes:** To achieve higher data rates and spectral efficiency, more inputs and OAM channels are needed. In this case, it might require specially designed couplers to the mode converter with more input waveguides (e.g., MMI or star coupler) [20, 86].
(c) **Polarization division multiplexing:** The mode converter might also be designed for dual-polarization mode multiplexing so that different inputs can excite the same modes on different polarizations [32].
(d) **Modal basis:** In this paper, the integrated devices are mainly focused on the 1-D OAM modal set. However, the general approach is potentially applicable to LG beams varying both indices [38, 87]. Moreover, different mode base sets could be valuable for different applications, for example, Hermite–Gaussian (HG) modes or linear polarized (LP) modes [38, 88], [89], [90].
(e) **Insertion loss:** Nonetheless, to accommodate the limited power budget in a wireless communication system, the insertion loss and the scalability are always tasks to improve. Possible solutions include (i) optimizing the partial-etch depth [32] and (ii) adding backside reflector layers [32].
